# Severe Acute Malnutrition Presenting With Scurvy: A Case Report

**DOI:** 10.7759/cureus.54506

**Published:** 2024-02-20

**Authors:** Varsha Premkumar, Renuka Jadhav, Sudhir Malwade, Shivani Kale

**Affiliations:** 1 Pediatrics, Dr. D. Y. Patil Medical College, Hospital & Research Centre, Dr. D. Y. Patil Vidyapeeth, Pune, IND

**Keywords:** vitamin c, vitamin c deficiency, child healthcare, diaphyseal fatty marrow, malnutrition, scurvy

## Abstract

Scurvy is a disease caused by a lack of vitamin C. It is a nutritional deficiency that is associated with multiple severe conditions. Although developed countries report these cases rarely now due to advancements in food and nutritional supplements, they are still prevalent in developing countries, albeit rare, because of poor nutritional status. Due to the lower prevalence of scurvy, diagnosis is delayed in the majority of cases and sometimes missed completely, which results in serious complications and unnecessary workups. Here, we present a rare case of a four-year-old female child with severe acute malnutrition (SAM) presenting with scurvy. The initial clinical signs showed SAM. X-ray and MRI of the left femur and knee were done to further evaluate the orthopedic parameters. Clinical presentation and radiographic imaging confirmed all the signs of scurvy. The patient was started on the Formula 75 (F-75) diet to address the severe malnutrition, and steady weight gain was observed.

## Introduction

Vitamin C is an essential dietary component as humans cannot synthesize it endogenously. The body's stores of vitamin C can run out in as little as one to three months if adequate amounts are not consumed through diet. Therefore, a balanced supply of vitamin C in the diet is critical. A deficiency of vitamin C in the diet leads to a nutritional disorder called scurvy. Scurvy is relatively rare; therefore, diagnosis could be delayed or missed entirely, leading to possibly adverse consequences. Several conditions, like weak connective tissues, joint pain, poor wound healing, bleeding gums, ecchymosis, and depression, have been attributed to prolonged scurvy [1-3]. Certain groups of people who consume food with limited varieties, have alcohol use-associated disorders, are elderly, and have poor nutritional status are more susceptible to vitamin C deficiency [4, 5]. Vitamin C is required for several basal-level functions in the body. It is important for the synthesis of collagen, which ultimately helps in the healing of wounds and the formation of bones as well [6]. It is also known to help in the synthesis of norepinephrine, a neurotransmitter, and enhance the absorption of iron in the stomach [7]. Apart from this, vitamin C also aids in the management of the inflammatory response by helping in the metabolism of prostaglandins [8]. Furthermore, vitamin C prevents cellular damage through its antioxidant properties.

## Case presentation

A four-year-old female child presented to the outpatient department with complaints of difficulty walking and an inability to sit for 15 days. The patient had a fall 15 days ago, and one week later, she gradually developed difficulty walking associated with bilateral pain. The patient had swelling over both lower limbs that was insidious in onset and gradually progressive in size. There were no complaints of fever, vomiting, abdominal pain, distention, difficulty in micturition, or seizures.

**Figure 1:**
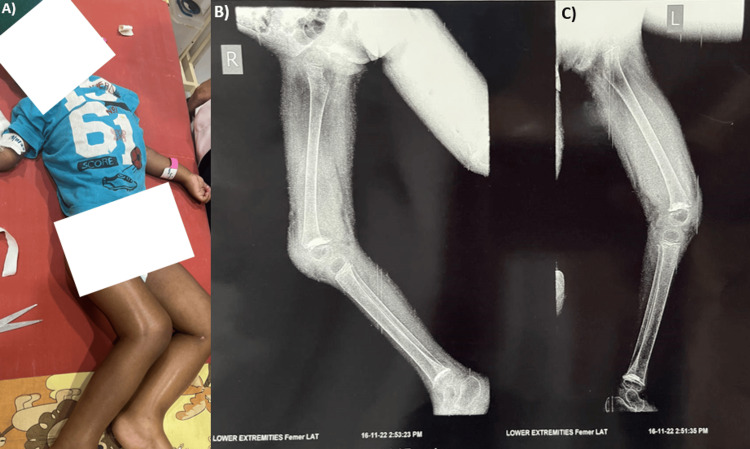
Clinical and radiology images of the patient A) A clinical picture of the patient presenting severe acute malnutrition; B) A lateral X-ray image of the right lower limb, confirming features of scurvy; C) A lateral X-ray image of the left lower limb, confirming features of scurvy

The child was born within a non-consanguineous marriage at full term without any neonatal intensive care unit (NICU) stay, with a family history of the mother having some acquired blindness. The patient was found to have not attained adequate weight and height for her age. There was no history of developmental defects, and the child was able to climb up and down the stairs. Social activities were normal; however, language was delayed and the child could only speak in bi-syllabic words. The initial clinical presentation of the patient also showed severe acute malnutrition (SAM) (Figure 1A). The patient was started on the Formula 75 (F-75) diet, a therapeutic liquid diet with an approximate energy density of 75 kcal/100 ml, and steady weight gain was observed. The catch-up diet, or F-100 diet, was started for the child two to seven days after starting the F-75 diet. Anthropometric parameters were analyzed and mostly found to be deficient according to age (Table 1).

**Table 1:** Anthropometric parameters of the patient SD: standard deviation

Anthropometric parameter	Value	Baseline value
Weight	7.1 kg	16 kg
Height	78.5 cm	101 cm
Mid-upper arm circumference (MUAC)	47.5 cm	13 cm
Weight for age	10 kg	-
Height for age	< -3 SD	-
Weight for height	< -3 SD	-
Head circumference	Between -2 SD and -3 SD	-

An orthopedic assessment was conducted, and the patient was scheduled for an MRI of the left femur and knee, accompanied by a Doppler examination, to investigate the possibility of deep vein thrombosis. The MRI of the left thigh was suggestive of subperiosteal hemorrhagic collection surrounding the femur, intraosseous hemorrhage in the distal metaphysis of the femur, and an altered marrow signal in the femoral shaft, indicative of likely edema. Additionally, findings included sclerosis, spur formation, widening, and irregularities in the distal femoral and proximal tibial metaphysis, resembling early diaphyseal fatty marrow pseudo-fractures. Subsequent X-ray examination exhibited characteristic features such as a ringed epiphysis (Wimberger ring), dense white line of Frankel, and corner sign (Figures 1B, 1C). The combined clinical presentation and radiographic evidence conclusively confirmed the diagnosis of vitamin C deficiency leading to scurvy.

## Discussion

The deficiency of vitamin C is found more commonly in areas of low socioeconomic status. Therefore, prevalence varies globally and ranges from 73.9% in northern India to 7.1% in the United States [9]. Newborns and infants are less likely to have a deficiency of vitamin C as it is supplemented by breast milk. As described previously, vitamin C is critical for collagen synthesis, bone formation, strengthening connective tissues, and several other body functions. An extensive review by Chin and Ima-Nirwana (2018) summarizes the role of vitamin C in maintaining bone health. The study, conducted in cell lines, a mouse model, and humans, concluded that vitamin C plays a vital role in osteoblast and osteoclast formation [6].

Hodges et al. (1971) have delineated the intricate pathophysiology of scurvy, explaining the interplay of vitamin C deficiency and the compromised state in SAM. This study has comprehensively elucidated the biochemical mechanisms underlying scurvy within the context of malnutrition [10]. Typically, the symptoms may go unnoticed or be misconstrued as symptoms of other conditions. In a study conducted by Kumar et al. in 2009, a case initially suggestive of juvenile idiopathic arthritis was examined orthopedically, and radiological assessments related to refractory arthritis surprisingly unveiled the presence of scurvy [11]. A retrospective study by Ratanachu-Ek et al. (2003) studied 28 cases of pediatric scurvy and found that 86% of the cases were previously misdiagnosed, primarily due to the presenting symptoms manifesting mobility disorders. However, the radiological examination revealed scurvy in the patients [12]. Bhutta et al. (2017) have described the distinct clinical features of SAM, emphasizing its widespread impact and severity. Scurvy with concurrent SAM is characterized by the specific creation of a unique diagnostic profile in these children [13-14].

In concurrence with the previous reports from the literature and the clinical presentation of the patient, an orthopedic evaluation was done through imaging, which confirmed vitamin C deficiency, which was scurvy with severe malnutrition.

## Conclusions

This case study highlights the fact that scurvy is a disease that exists, albeit rarely, even in developing countries. The diagnosis can be missed, yet it can be treated easily. Therefore, groups at high risk of malnutrition should be kept under a high level of suspicion. Timely intervention and management can relieve the patient of symptoms and suffering, and the patient can gradually recover completely.
